# The Scavenger Receptor MARCO Modulates TLR-Induced Responses in Dendritic Cells

**DOI:** 10.1371/journal.pone.0104148

**Published:** 2014-08-04

**Authors:** Haydn T. Kissick, Laura K. Dunn, Sanjukta Ghosh, Morris Nechama, Lester Kobzik, Mohamed S. Arredouani

**Affiliations:** 1 Department of Surgery, Beth Israel Deaconess Medical Center, Harvard Medical School, Boston, Massachusetts, United States of America; 2 Department of Environmental Health, Harvard School of Public Health, Boston, Massachusetts, United States of America; 3 Department of Medicine, Beth Israel Deaconess Medical Center, Harvard Medical School, Boston, Massachusetts, United States of America; 4 Department of Pathology, Brigham and Women’s Hospital, Harvard Medical School, Boston, Massachusetts, United States of America; Virginia Tech University, United States of America

## Abstract

The scavenger receptor MARCO mediates macrophage recognition and clearance of pathogens and their polyanionic ligands. However, recent studies demonstrate MARCO expression and function in dendritic cells, suggesting MARCO might serve to bridge innate and adaptive immunity. To gain additional insight into the role of MARCO in dendritic cell activation and function, we profiled transcriptomes of mouse splenic dendritic cells obtained from MARCO deficient mice and their wild type counterparts under resting and activating conditions. *In silico* analysis uncovered major alterations in gene expression in MARCO deficient dendritic cells resulting in dramatic alterations in key dendritic cell-specific pathways and functions. Specifically, changes in CD209, FCGR4 and Complement factors can have major consequences on DC-mediated innate responses. Notably, these perturbations were magnified following activation with the TLR-4 agonist lipopolysaccharide. To validate our *in silico* data, we challenged DC‘s with various agonists that recognize all mouse TLRs and assessed expression of a set of immune and inflammatory marker genes. This approach identified a differential contribution of MARCO to TLR activation and validated a major role for MARCO in mounting an inflammatory response. Together, our data demonstrate that MARCO differentially affects TLR-induced DC activation and suggest targeting of MARCO could lead to different outcomes that depend on the inflammatory context encountered by DC.

## Introduction

Scavenger receptors (SR) serve as molecular sensors on numerous cell types. Despite considerable progress in characterizing their function, many questions remain about their role in inflammatory and immune responses [1], [2]. Several clues indicate that SRs may influence cellular functions beyond pattern recognition and phagocytic clearance.

One SR, Macrophage Receptor with Collagenous Structure (MARCO), seems to have a number of immuno-modulatory functions. Mice deficient in MARCO suffer from exacerbated inflammatory response upon infection with Streptococcus, exposure to unopsonized particulate matter, ozone inhalation and ovalbumin challenge following sensitization, suggesting an anti-inflammatory role of MARCO [3]–[7]. Along the same lines, MARCO deficient (MARCO^−/−^) mice exhibited an early inflammatory response to influenza, characterized by rapid neutrophil influx to the lung, which appear to be beneficial in early resolution of influenza [8]. In contrast to these immuno-suppressive effects, in certain settings, MARCO is also important for immune activation. Silica induced mast cell activation, resulting in the production of TNF-α and reactive oxygen species (ROS) required MARCO and SR-AI/II [9]. In addition, activation of macrophages with CpG oligonucleotides resulting in IL-12 and nitric oxide (NO) production was dampened in MARCO^−/−^ mice, thereby indicating a pro-inflammatory role of MARCO [10]. This pointed to possible receptor cooperativity in directing downstream cellular events, and our work has previously suggested that MARCO engagement may be crucial for TLR9-mediated IL-12 production by macrophages in response to CpG [10]. In fact, recent evidence demonstrates that TLR signaling is finely tuned by the presence of co-receptors, notably scavenger receptors [11]–[14]. However, little is known regarding the role of MARCO in dendritic cells (DC), a cell type that bridges early innate immune response to activation of T lymphocytes. Genome-wide gene expression profiling of DC pulsed with tumor cell lysate revealed MARCO as the most upregulated gene [15]. Granucci and colleagues have shown that MARCO mediates cytoskeletal rearrangements promoting dendritic lamellopodia [16], a finding that is in line with later studies showing MARCO inhibits DC migration, with pathophysiological consequences on allergic asthma and cancer immunotherapy [3], [17]. These observations provided sound rationale to explore the role MARCO in DC activation following TLR engagement. Our results suggest a major role for MARCO in regulating TLR-induced inflammatory response and provide context for several previously reported functions of MARCO. Taken together, our findings highlight TLR subclass-specific role for MARCO in modulating DC function and broadens the spectrum of MARCO contribution to the regulation of immunity and inflammation.

## Materials and Methods

### Animals

Eight- to twelve-week-old mice genetically deficient in MARCO (MARCO^−/−^) were described previously [3], [6], [7]. Age- and sex-matched C57BL/6 wild-type (WT) mice purchased from Charles River Laboratories (Wilmington, MA) were used as controls. All mice were housed in pathogen-free conditions, and all experimental procedures involving animals were approved by the Institutional Animal Care and Use Committee at Beth Israel Deaconess Medical Center.

Discomfort and injury to animals was limited to that which was unavoidable in the conduct of scientifically valuable research. All personnel performing the animal procedures/manipulations/observations described in this protocol are technically competent and have been properly trained to ensure that no unnecessary pain or distress was caused to the animals as a result of the procedures/manipulations. Mice were euthanized using CO_2_ inhalation in a CO_2_ SMART BOX.

### Cell lines

The DC2.4 cell line, derived from C57BL/6 bone marrow [18], was kindly provided by Dr. Kenneth Rock (University of Massachusetts Medical Center, Worcester, MA). Cells were grown in complete media comprised of DMEM, supplemented with 10% FBS, 10 mM HEPES, 2 mM L-glutamine and 50 µg/ml gentamicin. DC2.4 cells were maintained at 37°C in a humidified incubator with 5% CO_2_. Cells were maintained via weekly passage and utilized for experimentation at 60–80% confluency.

### Isolation of splenic dendritic cells

Spleens of untreated adult mice were digested using Spleen Dissociation Medium (Cat #07915, STEMCELL Technologies). Dendritic cells were isolated by positive selection from the using the EasySep Mouse CD11c Positive Selection Kit (Cat #18758, STEMCELL Technologies). These DC are CD11c positive and more than 90% of them express MHC-II and the costimulatory receptors CD80 and CD86.

### *In vitro* activation of DC with toll-like receptor agonists

Three DC pools were obtained from both MARCO^−/−^ and age- and gender-matching control C57BL/6 mice by purifying spleen DC from 5–6 animals per pool. DC from each pool were cultured overnight at 10^6^/ml in 24-well plates in the presence or absence of TLR agonists (Invitrogen) LPS (100 ng/mL), PAM3 (Pam3CSK4, 1 µg/mL), R848 (350 nM), POLYIC (50 µg/mL), CPG (0.5 µM) and FLAST (20 µg/mL). All ligands were culture-tested and endotoxin free. DC2.4 cells were treated similarly.

### RNA Extraction

Total RNA was isolated from treated and untreated cells using Trizol reagent. RNA was quantified by NanoDrop ND-1000 spectrophotometer, and quality was evaluated with Agilent RNA 6000 NanoChip and the 2100 Bioanalyzer, with 28S/18S ratios and RIN determined by 2100 Expert software.

### Gene Expression Profiling

Gene expression was assessed using Affymetrix (Santa Clara, CA) GeneChip *Mouse* Genome *430* 2.0 arrays. 15 µg cRNA was fragmented and hybridized to arrays’ according to the manufacturer’s protocols as described previously [19]. The quality of scanned array images were determined on the basis of background values, percent present calls, scaling factors, and 3′/5′ ratio of β-actin and GAPDH. Data were extracted from CEL files and normalized using RMAexpress (http://rmaexpress.bmbolstad.com/) and annotated using MeV software (http://www.tm4.org/mev.html). Differentially expressed genes between different conditions were determined using a fold change threshold of 2. “The data generated have been deposited in NCBI’s Gene Expression Omnibus and are accessible through GEO Series accession number GSE55068 (http://www.ncbi.nlm.nih.gov/gds/?term=GSE55068)”.

Data showing MARCO expression in response to TLR ligation in bone marrow-derived DC were extracted from the gene expression dataset GSE17721 [20]. CEL files were downloaded from the NCBI Gene Expression Omnibus (GEO) and processed for normalization using the RMAexpress tool and gene annotation using the MeV software.

### Pathway and Functional Analysis

Genes that showed a fold change in expression of at least 2 were uploaded onto the Ingenuity Pathways Analysis tool (Ingenuity Systems, http://www.ingenuity.com). IPA applications were used to generate and assess statistically relevant biofunctions, canonical pathways, networks and changes in transcription factor status associated with the differentially expressed gene profiles extracted from the transcriptome data.

Pathway and Functional analyses of the differentially expressed genes were performed using the commercial systems biology oriented package Ingenuity Pathways Analysis (www.ingenuity.com). Ingenuity Pathway Analysis Tool was used to calculate the p-value with Fisher’s Exact Test for each pathway and functions. The p-value measures the likelihood of random chance for the observed association between a specific pathway/function in the dataset, by also considering the total number of Functions/Pathways/Lists of eligible genes in the dataset and the Reference Set of genes (those which potentially could be significant in the dataset). In case of interactive networks, all the identified genes were mapped to genetic networks available in the Ingenuity database and were ranked by the score. The Score (−log P value) is calculated using Fisher’s Exact Test and indicates the likelihood a gene will be found in a network due to random chance. For example, if a network achieves a score of 2, it has at least 99% confidence of not being generated by chance alone.

### Transcription Factor and miRNA Profiling

Ingenuity’s Upstream Regulator Analysis is a tool that allows prediction of the activation status of various regulators, including transcription factor and micro-RNAs, based on genome-wide differentially expressed gene signature. This tool predicts which transcriptional regulators and micro-RNAs are involved and whether they are likely to be *activated* or *inhibited*. The activation status of a given regulator is predicted through a calculated activation z-score where z>2 predicts activation and z<−2 predicts inhibition.

### Quantitative Real-Time PCR (qRT-PCR)

Validation of differentially expressed genes and genes belonging to specific pathways and functions was performed by RT-PCR. 200 ng of high quality RNA samples were reverse transcribed to first strand cDNA and 1 µl cDNA was used for each RT-PCR reaction. Samples were performed in triplicates. SYBR Green PCR Master Mix (Applied Biosystems, Foster City, CA) was used for two-step real-time RT-PCR analysis on an Applied Biosystems StepOnePlus Real Time PCR instrument. Primers’ sequences were designed using the rpimer3 tool (http://bioinfo.ut.ee/primer3-0.4.0/primer3/). Expression value of the targeted gene in a given sample was normalized to the corresponding expression of GAPDH. The 2^–ΔΔCt^ method was used to calculate relative expression of the targeted genes.

### Statistical analysis

Student’s *t* test (unpaired, two-tailed) was calculated using GraphPad Prism to determine significance levels between groups and treatments for all RT-PCR measurements. Data are presented as mean ± SEM or SD. Differences were considered significant when *p*<.05.

### Ethics Statement

All experimental procedures involving animals were approved by the Institutional Animal Care and Use Committee at Beth Israel Deaconess Medical Center. We assure that discomfort and injury to animals was limited to that which was unavoidable in the conduct of scientifically valuable research and that analgesic, anesthetic, and/or tranquilizing drugs were used where indicated and appropriate to minimize pain and/or distress to animals. All personnel performing the animal procedures/manipulations/observations described in this protocol are technically competent and have been properly trained to ensure that no unnecessary pain or distress was caused to the animals as a result of the procedures/manipulations.

## Results

### Basal and induced MARCO expression in bone marrow-derived and mature splenic dendritic cells

**Figure 1:**
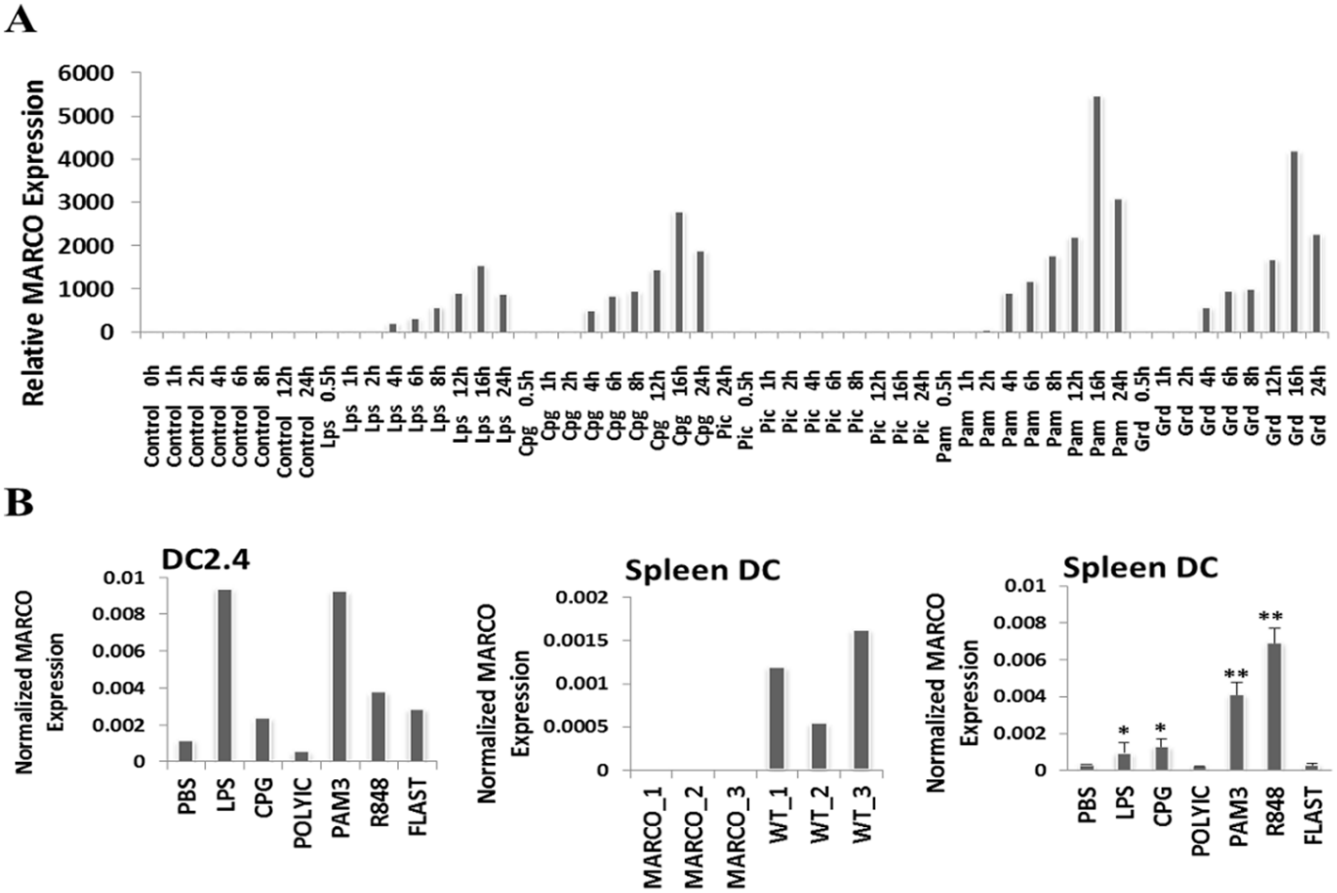
Expression of MARCO receptor in splenic and bone marrow-derived DC. (**A**) MARCO gene expression was determined in BMDC at various time points following treatment with various TLR agonists. Raw data from gene expression dataset GSE17721 [20] were analyzed to extract MARCO expression values. Data were processed for normalization using the RMAexpress tool and gene annotation using the MeV software. (**B**) MARCO expression as determined by RT-PCR is shown in TLR agonist-activated DC2.4 cell line (left panel), splenic DC from WT and MARCO^−/−^ DC from 3 individual mice (middle panel), and TLR agonist-activated splenic DC (right panel). GAPDH expression was used for normalization. Data shown as Mean ± SD from triplicates. *P<.05; **P<.01.

To gain insight into the regulation of MARCO expression in response to DC activation, we analyzed publicly available gene expression profiling data [20]. I*n silico* analysis revealed similar expression kinetics in mouse bone marrow-derived dendritic cells (BMDC) in response to all TLR agonists investigated. In this data set, MARCO mRNA was detectable starting at 4 h and peaked at 16 hours following exposure to agonists of TLR1/2, TLR4, TLR7/8 and TLR9, but not TLR3 (Figure 1A). We extended these findings experimentally, and found that the DC2.4, a mouse BMDC cell line, did not increase expression of MARCO in response to the TLR-3 agonist PolyIC, but expression of MARCO was increased by the other TLR agonists (Figure 1B, Left Panel). Mature splenic DC from adult C57BL/6 mice, purified using positive selection and confirmed by flow cytometry to express the CD11c, MHC-II and CD80 markers, express low, yet detectable levels of MARCO in the absence of stimulation as detected by RT-PCR, while MARCO^−/−^ mice show no expression (Figure 1B, Middle Panel). Following *in vitro* challenge with TLR agonists, PolyIC and Flagellin, these cells failed to induce MARCO expression, while LPS, CpG, Pam3 and R848 induced significant levels of expression, compared to the PBS-treated cells (Figure 1B, Right Panel). Together these results indicate that expression of MARCO is induced in numerous DC models by certain TLR agonists.

### Differential gene expression in WT and MARCO^−/−^ DC indicate altered phenotype and response characteristics

**Figure 2:**
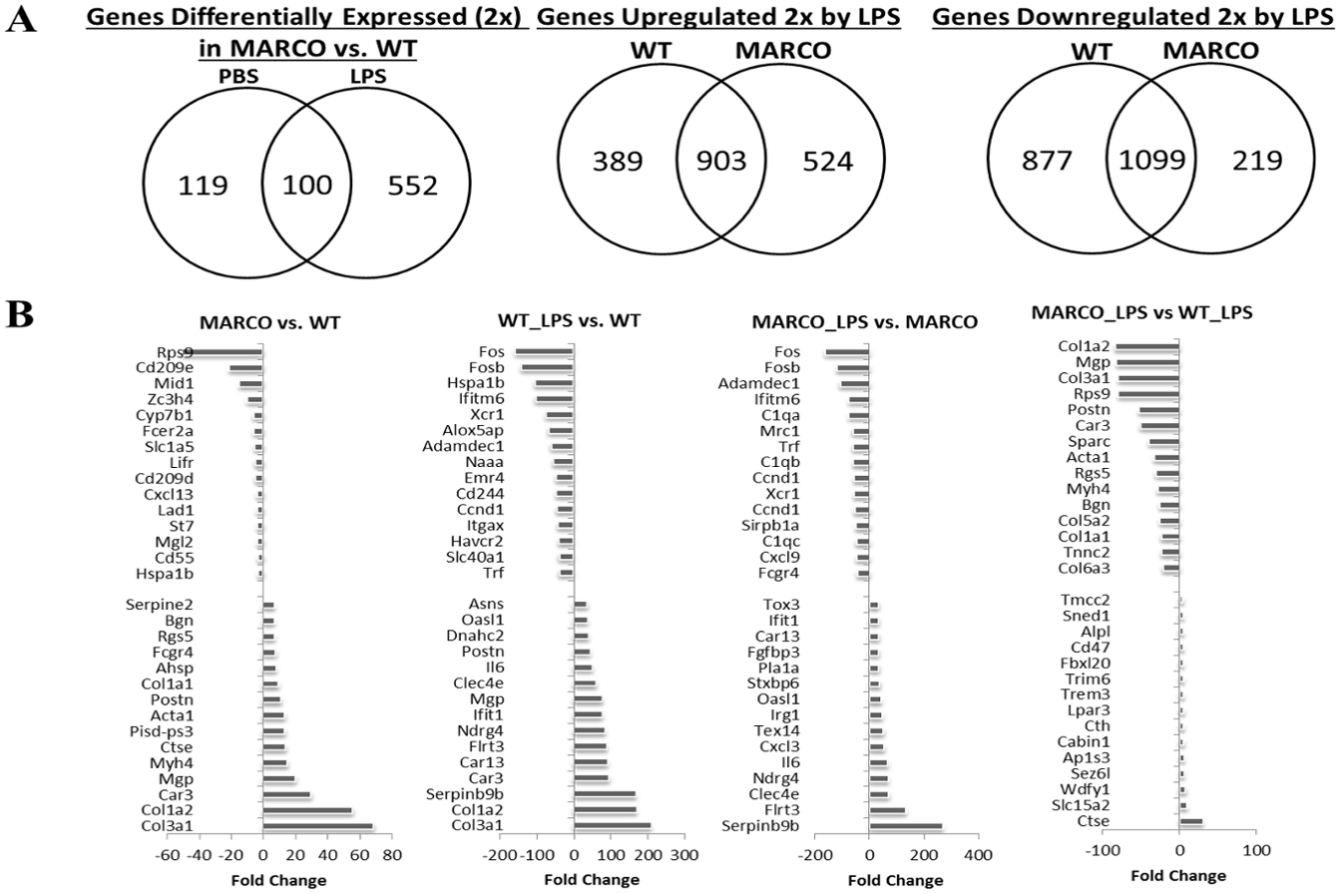
Differentially expressed genes in WT and MARCO^−/−^ DC cells. High purity DC preparations were isolated from splenocytes from 5–6 mice per group by positive selection with CD11c antibody and incubated overnight in media containing PBS or LPS (100 ng/ml). Total RNA was extracted and subjected to gene expression profiling. (**A**) Venn diagrams showing the numbers of genes that differ in expression by a factor of at least 2 between WT and MARCO^−/−^ DC without and with LPS (left diagram), and numbers of genes that are differentially upregulated (middle diagram) or downregulated (right diagram) in WT and MARCO^−/−^ DC following LPS exposure. (**B)** Top 15 differentially expressed genes that characterize MARCO vs. WT, WT_LPS vs. WT, MARCO_LPS vs. MARCO, and MARCO_LPS vs. WT_LPS. Data shown represent fold change of gene expression.

To investigate the effect of basal MARCO expression in splenic DC, we first profiled genome-wide gene expression of resting DC to identify inherent differences between WT and MARCO-deficient cells. A total of 219 genes showed differential expression by at least 2-fold between WT and MARCO^−/−^ DC (Figure 2A). Within these data, we found changes in genes related to the extracellular matrix and plasma membrane components. Highly significant upregulation of multiple collagen transcripts type I, II, IV, V and VII were noted in MARCO^−/−^ cells. Upregulation of matrix Gla protein (20.3-fold), osteoblast specific periostin (11-fold), osteonectin Sparc (6-fold), BMP2, fibronectin 1 (5.9-fold), and fibrillin (2.6-fold), lectin (3.4-fold), tissue inihibitor of matrix metalloproteinase inhibitor 1 (2.6-fold) and MMP2 (2.1-fold) were observed in MARCO^−/−^ over WT cells. Likewise, an interesting repertoire of transcripts of plasma membrane proteins was upregulated in MARCO^−/−^ at steady state, namely CD16a (IgG FcIIIa, 8-fold), CD160 (2.5-fold), integrin beta 5 (2.5-fold), endothelial cell adhesion molecule (2.1-fold), and caveolin 1 (2.3-fold). There was decreased expression of CD209 (DC-SIGN) in MARCO^−/−^ cells by nearly 4.5 folds, and reduced CD55 (complement regulated gene) by nearly 3-fold (Figure 2B).

**Figure 3:**
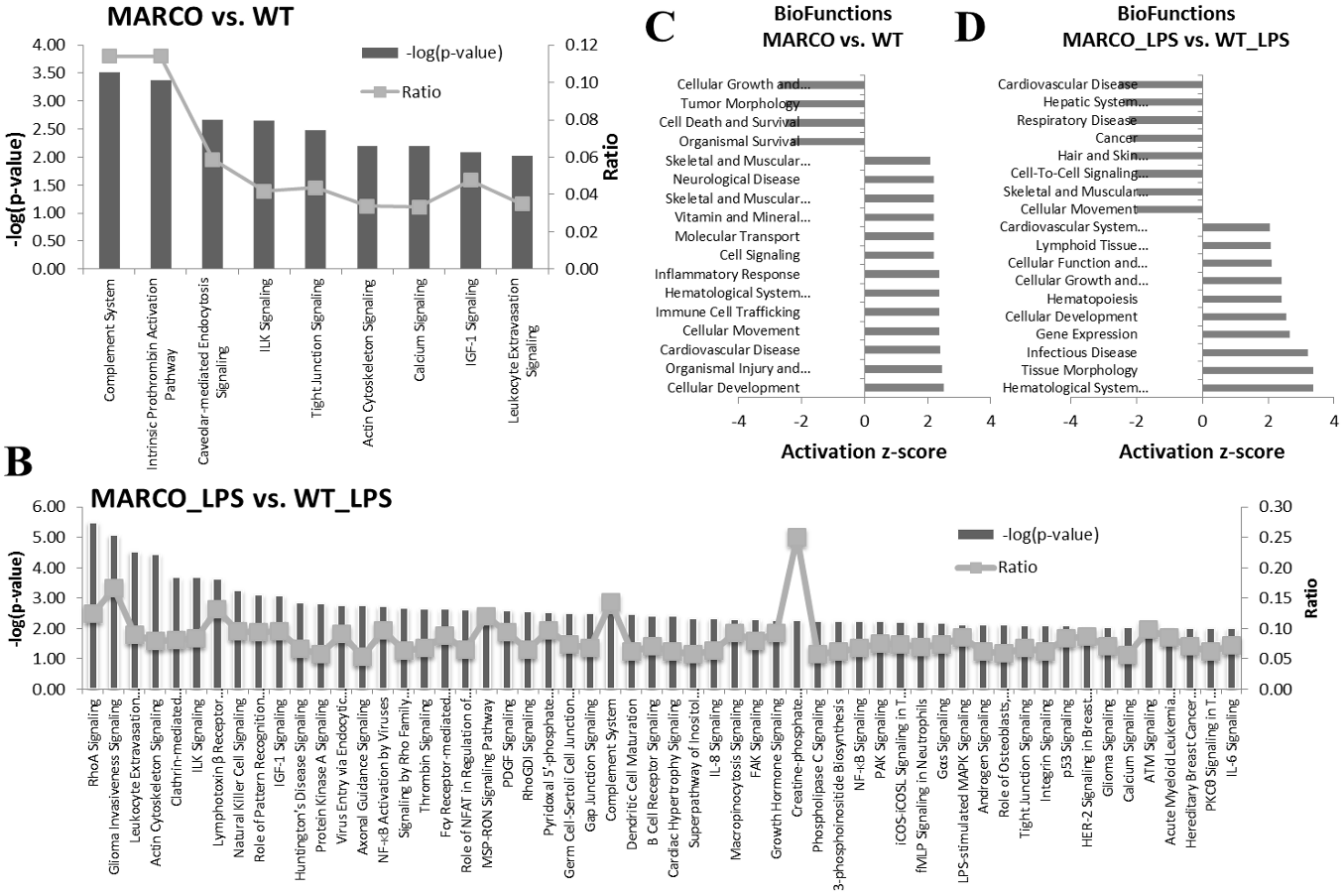
Major DC Signaling Pathways and Functions are affected by MARCO. Sets of differentially expressed genes (fold change of 2 or higher) between different DC conditions were uploaded onto Ingenuity Pathway Analysis and corresponding signaling pathways were predicted. (**A**) MARCO vs. WT. (**B**) MARCO_LPS vs. WT_LPS. Statistical significance was set at −log P = 2 (Left Y Axis). The Ratio on the right Y axis represents the fraction of genes that are differentially expressed in our dataset that fall within a specific pathway out of the total number of genes that contribute to that pathway. Similarly, Biofunction analysis was performed for MARCO vs. WT (**C**) and MARCO_LPS vs. WT_LPS (**D**). Statistical significance was set at activation z-score = 2. Scores higher than 2 indicate activated functions, whereas scores lower than −2 indicate inhibited functions.

The set of 219 differentially expressed genes were further organized into functional groups of biological functions and signaling pathways using the Ingenuity Pathway Analysis package (www.ingenuity.com). The Complement pathway was the most significantly altered among the differentially expressed genes between MARCO^−/−^ and WT DC. Significant differential expression was also observed in genes involved in caveolar-mediated endocytosis, tight junction signaling, cytoskeleton signaling, leukocyte extravasation signaling, and calcium signaling, among others (Figure 3A).

### TLR4 ligation widens gene expression gap between WT and MARCO^−/−^ DC

We next evaluated the impact of MARCO deficiency on DC activation, using LPS as a surrogate for Gram(−) bacterial infection or adjuvant-supplemented vaccination. DC were treated with LPS or vehicle overnight. The time point was chosen for optimal induction of most LPS inducible genes as deduced from available gene expression profiling data, as well as to specifically evaluate the relatively early response of DC to LPS stimulation in presence and absence of MARCO. Following this activation, 652 gene transcripts were differentially expressed by 2-fold or greater in MARCO^−/−^ over WT, nearly 3 times higher when compared to the 219 genes that changed in the absence of LPS (Figure 2A, Left Venn Diagram), including 100 shared transcripts. The 652 gene set includes many genes that have been shown to play key roles in DC biology (Figure 3B, 3C & 3D). Pathway analysis of differentially expressed genes revealed deregulations in Rho A signaling pathway, leukocyte extravasation signaling, actin cytoskeleton signaling, clathrin-mediated endocytosis signaling, pattern recognition receptor function, PKA signaling, NF-kB activation and signaling, Rho family GTPases signaling, FCγR-mediated phagocytosis, complement system, DC maturation, LPS-induced MAPK signaling, integrin signaling, IL-6 signaling, among others (Figure 3B). It is noteworthy that genes related to pattern recognition receptors in bacterial infection were also within this category of highest significance, supporting the validity of the comparison. Furthermore, comparison of the biofunctional analysis for the non-stimulated versus stimulated MARCO^−/−^ over WT revealed interesting features, presented in Figures 3C & 3D. Functions identified as cellular movement, immune cell trafficking, and inflammatory response are predicted to be activated in unstimulated MARCO^−/−^ DC. Of note, the cluster of genes responsible for cell movement were upregulated in MARCO^−/−^ cells over WT cells in unstimulated conditions, whereas WT cells showed upregulation of this class of genes over the MARCO^−/−^ following LPS exposure, implying that MARCO is involved in LPS-induced cell migration.

Additionally, we enumerated clusters of genes that are differentially expressed in WT and/or MARCO^−/−^ DC under activating conditions. Our data suggest that the presence of MARCO in DC (i.e. WT phenotype) correlates with upregulation and downregulation of 389 and 877 genes, respectively, while its absence results in upregulation and downregulation of 524 and 219 genes (Figure 2A, Middle and Right Venn Diagrams). Together, this data suggests an involvement of MARCO in LPS/TLR4-induced regulation of 2009 genes.

### MARCO confers a distinct transcriptional factor profile to DC regardless of their activation state

**Figure 4:**
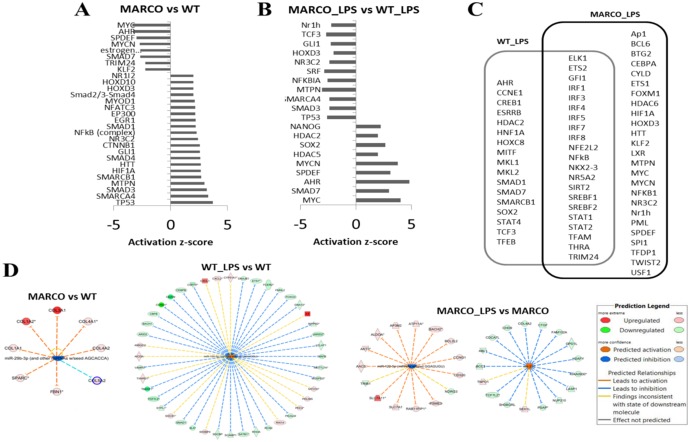
Comparison of Upstream Regulator status between WT and MARCO^−/−^ DC. Differentially expressed genes (fold change of 2 or higher) were processed through Ingenuity Pathway Analysis to predict the downstream regulators whose activation status was affected by the absence of MARCO in resting cells (**A**) or LPS-challenged cells (**B**). The Venn diagram in (**C**) shows the transcription factors that respond to LPS in WT (WT_LPS) and MARCO^−/−^ (MARCO_LPS) DC. Transcription factors that reached the significant activation z-score of −2 or +2 are shown. (**D**) Shown are representative microRNAs that reached the significant activation z-score of −2 or +2. The IPA tool predicts a microRNA to be activated when enough differentially downregulated genes fall among the targets for this microRNA. The inhibition status is attributed when the opposite occurs.

The dramatic differences in gene expression between MARCO sufficient and deficient DC suggests major alterations take place at the level of transcription factors. We used Ingenuity’s Upstream Regulator Analysis tool to unravel transcription factors that had significant perturbations. As evident in Figure 4, presence and absence of MARCO in DC resulted in distinct transcription factor activation status in both steady state (Figure 4A) and following LPS activation (Figure 4B). Interestingly, NF-κB1A, an inhibitory member of proinflammatory transcription factor NF-κB family, is down-regulated in MARCO^−/−^ cells following LPS stimulation, in comparison to WT. This could imply decrease in the regulatory component I-κB, and conversely, an increase in the pro-inflammatory transcription factor engagement within the MARCO^−/−^ cells. This appears to be the case indeed in the non-stimulated MARCO^−/−^ cells where NF-κB complex gains prominence over WT cells.

When looking at the transcription factor subsets that are affected in WT and MARCO^−/−^ DC following LPS activation, one can see genotype-specific profiles, with 17 factors affected exclusively in WT, 25 factors affected exclusively in MARCO^−/−^, and 21 overlapping factors that include 6 members of the Interferon Regulatory Factor (IRF) family (Figure 4C).

Next, we used Ingenuity Pathway Analysis to predict the status of microRNAs that regulate MARCO-driven differential gene expression. MicroRNAs are important regulators that modulate gene expression and thereby influence effector cell function of immune cells, including DC [21]. In the absence of MARCO, and under resting conditions, one single perturbation was predicted that inhibits miR-29b-3p and potentially other micro-RNAs that share the same target specificity. These micro-RNAs regulate genes that were down-regulated in MARCO-deficient DC, including COL1A1, COL1A2, COL3A1, COL4A1, COL4A2, COL5A2, FBN1 and SPARC (Figure 4D). When WT DC were challenged with LPS, 10 micro-RNAs were predicted to be activated (z score >2, data not shown), including miR-155-5p illustrated in Figure 4D. MARCO^−/−^ DC, in contrast, showed only 2 changes, with activated miR-210 and inhibited miR-122-5p in response to LPS (Figure 4D).

### MARCO deficiency leads to perturbations in the TGF-β pathway

**Figure 5:**
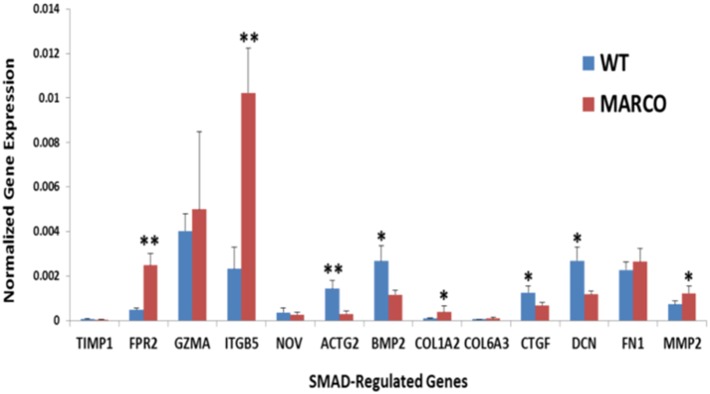
Involvement of MARCO in the TGF-β signaling pathway. Expression of differentially expressed genes in our dataset that are known to be regulated through by SMAD transcription factors within the TGF-β signaling pathway was measured by RT-PCR in resting WT and MARCO^−/−^ DC. *P<.05, **P<.01. Data show the mean ± SD of 3 WT and 3 MARCO^−/−^ samples where each sample represents a pool of 3 splenocyte preparations.

Equally interesting is the fact that the Smad family of transcription factors was heavily represented as differentially altered in the control sets (Figure 4). In our experiments, unstimulated MARCO^−/−^ cells showed activated Smad-2/3-Smad-4 axis, with significant enhancement of Smad-1, Smad-4 and Smad-3, and a concomitant inactivation of Smad-7 (Figure 4A). Conversely, Smad-7 is activated in LPS-stimulated MARCO^−/−^ cells in comparison with stimulated WT cells (Figure 4B). Therefore, we sought to further validate this *in silico* prediction. We chose a panel of Smad-3- and Smad-7-responsive genes through IPA analysis and compared their expression levels by RT-PCR in the presence or absence of MARCO in non-activated cells. The data show a significant increase in expression of FPR2, ITGB5, COL1A2 and MMP2 that occurred in MARCO^−/−^ DC, whereas a decrease in ACTG2, BMP2, CTGF, and DCN is noted (Figure 5).

### MARCO exerts differential effects on TLR-induced DC activation

Our *in silico* analysis revealed inherent differences in gene expression between WT and MARCO^−/−^ DC, and clearly demonstrates an amplifying role for LPS activation on these differences. This suggests an important role for MARCO on DC in the context of proinflammatory insults such as LPS challenge. In light of these findings and the previously reported interactions of MARCO with members of the TLR family on macrophages, we sought to examine the role of MARCO in DC responsiveness to a panel of TLR agonists. This *in vitro* model, where synthetic surrogates of known natural TLR ligands are used to challenge DC, closely recapitulates exposure to bacterial, viral and fungal infection, and equally emulates DC exposure to TLR-targeted adjuvants in the context of active immunization.

**Figure 6:**
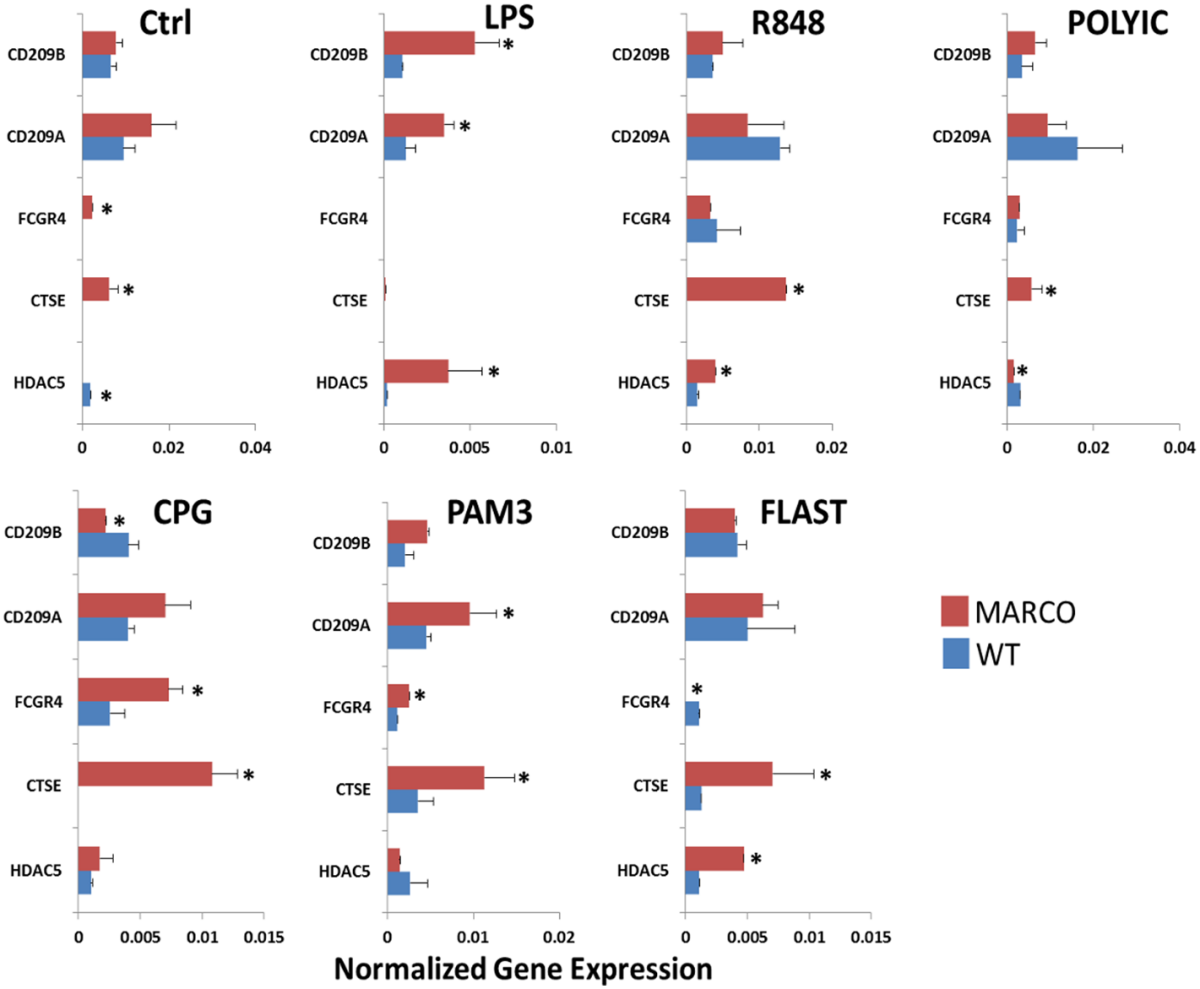
Validation of highly differentially expressed genes between WT and MARCO^−/−^ DC in response to TLR challenge. DC were cultured overnight in the absence and presence of different TLR agonists. RT-PCR was performed to measure gene expression. *P<.05 for MARCO^−/−^ **vs.** WT DC. Data show 3 WT and 3 MARCO^−/−^ samples where each sample represents a pool of 3 splenocyte preparations.

DC were cultured overnight in the absence or presence of TLR agonist doses that were shown to induce IL-6 expression under similar experimental conditions (data not shown). RT-PCR was first used to quantify the expression level of a set of genes that were selected from our transcriptome profiling data based on the magnitude of their differential expression. In the latter category, the cell surface receptor DC-SIGN (CD209) offered an interesting trend across the different TLR agonist treatments. At steady state, WT and MARCO^−/−^ DC expressed similar amounts of CD209A and CD209B mRNA transcripts. Upon activation, differences between WT and MARCO^−/−^ DC were observed in response to LPS, CPG and PAM3. FCGR4, also known as Fc Receptor-like 3 (Fcrl3) and CD16-2, is absent in WT DC but present at a low level in MARCO^−/−^ DC. Upon activation, its differential expression varies between agonists. Similar to FCGR4, Cathepsin E (CTSE) is only present in non-activated MARCO^−/−^ DC. Interestingly, while the CTSE gene in MARCO^−/−^ DC responded to all TLR agonists to various extents, WT DC only responded to PAM3 and FLAST. Conversely, Histone deacetylase 5 (HDAC5) is only expressed in WT DC and its response to TLR agonists is overall weak and variable (Figure 6).

**Figure 7:**
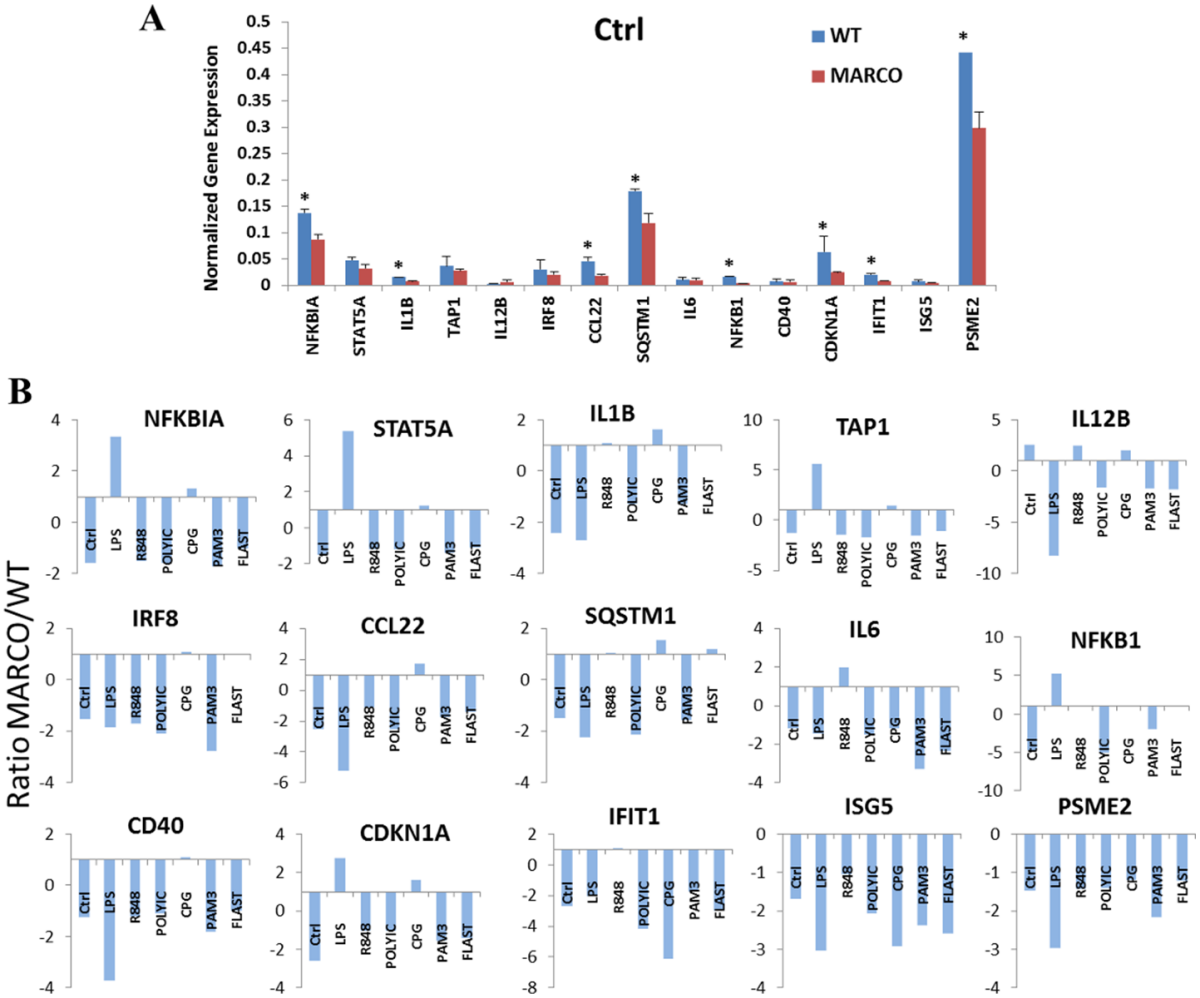
Differential expression of immune and inflammatory marker genes between WT and MARCO^−/−^ DC in response to TLR agonist challenge. DC were cultured overnight in the absence and presence of different TLR agonists. RT-PCR was performed to measure gene expression. (A) Basal expression in WT and MARCO^−/−^ DC in the absence of TLR ligation. *P<.05. Data show the mean ± SD of 3 WT and 3 MARCO^−/−^ samples where each sample represents a pool of 3 splenocyte preparations. (B) Gene expression ratio for MARCO/WT was calculated to reveal the magnitude of MARCO’s contribution for each individual gene across all TLR agonists.

RT-PCR quantitation was next performed to assess a panel of immune and inflammatory marker genes. We found that all the genes tested were expressed at a relatively low level in non-activated DC regardless of the presence of MARCO, albeit with a tendency for slightly higher expression in the WT (Figure 7A). Interestingly, the challenge of WT and MARCO^−/−^ DC with TLR agonists elicited responses that widely varied in magnitude depending on the agonist and target gene. This trend is more evident when data are plotted as a MARCO/WT expression ratio to highlight the impact of MARCO deficiency (Figure 7B). Most ratios are lower than 1, indicating that MARCO deficiency causes a decrease in gene expression, suggesting thus a positive role for MARCO in regulating expression of these genes.

**Figure 8:**
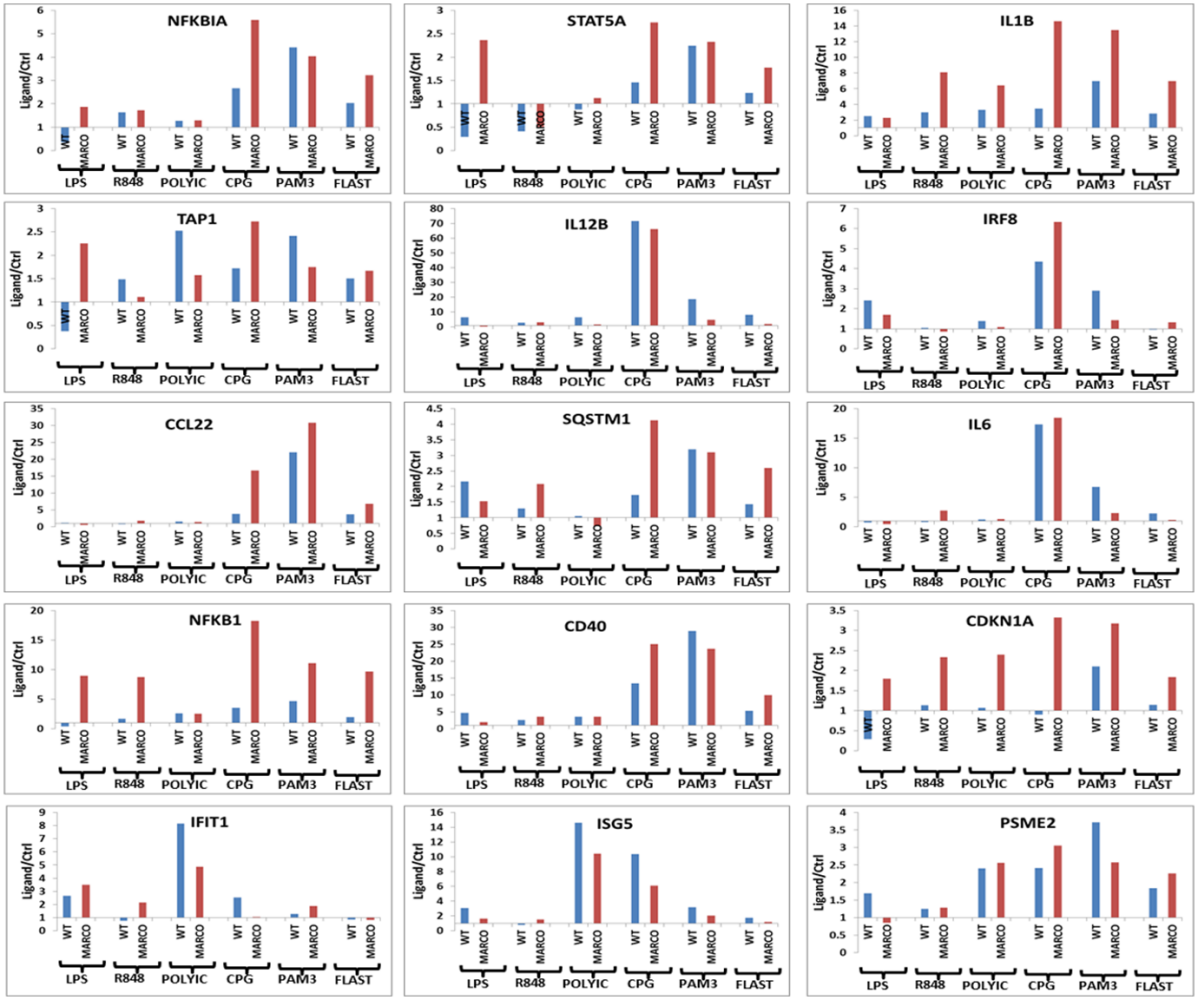
Impact of MARCO on DC responsiveness to different TLR agonists. RT-PCR data from Figure 7 was plotted using the Ligand/Ctrl ratio to reveal the contribution of the presence and the impact of the absence of MARCO on TLR-induced inflammatory gene signature in WT and MARCO^−/−^ DC, respectively. The Ligand/Ctrl Ratio was calculated for each gene to allow comparisons between WT and MARCO^−/−^ DC across all TLR agonists.

However, because inherent differences in expression of several genes were observed in the absence of agonists (Figure 7A), we sought to determine the effect of MARCO on DC responsiveness to each TLR agonist. To this end, we calculated the ratio of ligand-induced expression value to control expression value (i.e. in the absence of ligand) for each individual gene in both WT and MARCO^−/−^ DC. Using this ratio led to a number of interesting observations; First, LPS seems to augment or suppress gene expression depending on the gene, with the suppression preferentially affecting WT DC. Second, all other TLR agonists enhance expression of all genes, with the exception of STAT5A, regardless of the MARCO status. Last, while overexpression of some genes, e.g. STAT5A, IL1B, CCL22, NFKB1, and CDKN1A, is more prominent in MARCO^−/−^ DC, other genes like IL12B and IRF8 are preferentially overexpressed in WT DC (Figure 8).

### MARCO effects are not due to differential TLR expression

**Figure 9:**
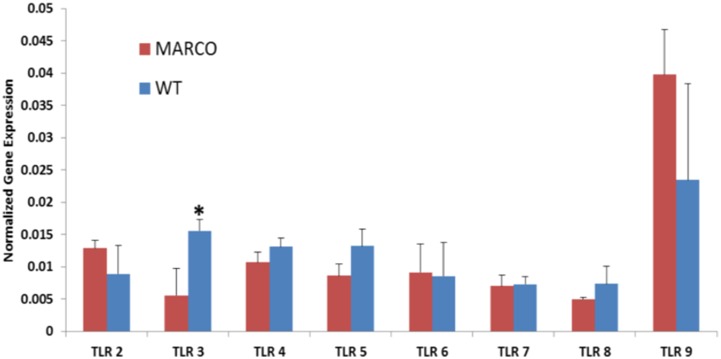
Impact of MARCO on TLR gene expression in DC. TLR2-9 gene expression was determined in unstimulated splenic WT and MARCO^−/−^ DC. Data show the mean ± SD of 3 WT and 3 MARCO^−/−^ samples where each sample represents a pool of 3 splenocyte preparations. *P<.05. GAPDH expression was used for normalization.

Available data suggest cooperativity between scavenger receptors and TLRs [11]. Hence, differences in expression of TLRs on the surface of DC in the absence and presence of MARCO might skew this cooperativity. Therefore, to ascertain that the observed effects are not due to intrinsic differences in TLR expression between WT and MARCO^−/−^ DC, we quantitated RNA transcripts for TLR1-9 in untreated DC from both genotypes. Interestingly, TLR-3 is the only member of the TLR family that is differentially expressed, showing a significant decrease in the absence of MARCO, whereas a trend of increased TLR-2 and TLR-9 expression in MARCO^−/−^ DC did not achieve statistical significance (Figure 9).

## Discussion

In the present study we show that spleen DC express the receptor MARCO and MARCO expression is inducible through TLR ligation. Interestingly, among all TLR agonists we tested, Poly-IC is the only one that failed to trigger MARCO expression, suggesting TLR-3-induced signaling is not involved in regulating MARCO expression in spleen DC. A similar finding was reported previously using bone marrow-derived dendritic cells [20], [22] and macrophages [23]. Because TLR-3-mediated signaling, unlike signaling through other TLRs, does not require the adaptor MyD88 [24], our finding also suggests a key role for MyD88 in inducing MARCO expression.

At the genome-wide gene expression level, MARCO expression on resting DC correlates with marked divergences between WT and MARCO^−/−^ DC. Interestingly, these divergences become even more prominent following LPS treatment, suggesting an important role for MARCO in TLR4-induced signaling. Furthermore, significant differences are observed in expression of pro-inflammatory markers in response to several TLR agonists, suggesting MARCO’s contribution to cell signaling might be a critical component of a feedback loop that is common to all TLRs on DC.

While many observations emerging from our *in silico* analysis and RT-PCR validation deserve careful interpretation, three of them may be of special interest and therefore will be addressed here. First, particularly important is our observation that alterations in the activation status of Smad proteins, the main effector regulators of the TGF-β pathway, are taking place in MARCO^−/−^ DC under both resting and activating conditions. Notably, LPS challenge reverses the activation state of SMAD proteins observed in resting cells. These predicted dysregulations in the TGF-β signaling pathway were reflected in significant expression changes of many TGF-β-regulated genes, as evidenced by our RT-PCR measurements. This observation is of paramount importance because TGF-β pathway is crucial in determining DC phenotype and T cell activation [25]. TGF-β prevents autoimmunity by maintenance of immature DC in a tolerogenic state. The tolerogenic effect of immature DC is due to soluble TGF-β secreted by Regulatory T cells [26]. Additionally, TGF-β secreted by tumor cells and tumor-associated macrophages tolerizes DC in the tumor and draining lymph nodes thus hampering anti-tumor immunity [27], a powerful mechanism of immune tolerance to tumors that could be reversed by TGF-β or TGF-β receptor blockade [28]. Second, MARCO seems to exhibit an inhibitory effect resulting in lower production of IL-12β and CDKN1A in WT DC. IL-12 plays a crucial role in Th1 differentiation [29], thus driving anti-viral and anti-tumor adaptive responses [30]. Interestingly, the effect of MARCO on IL-12β expression might also extend to the production of IL-23. IL-12 and IL-23 share the IL-12β chain [31]. IL-12 promotes Th1 immunity and IL-23 promotes Th17 immunity, and it has recently become apparent that the balance between IL-12 and IL-23 is very important in immune regulation (Reviewed in ref. [32]). In a recent study, Komine et al. generated a new MARCO-deficient mouse to address the role of MARCO in DC [33]. This study showed low expression of MARCO in resting BMDC, with a significant increase following challenge with LPS or tumor lysate. It also showed an increased motility of MARCO^−/−^ DC. However, there were no differences in the release of IL-12, IL-10 or TNF-α between WT and MARCO^−/−^ BMDC following LPS treatment, which might reflect one of the intricate differences between splenic and bone-marrow derived DC. Finally, while the effect of MARCO on the responsiveness of DC to agonists that recognize cell surface TLRs (TLR-2, 4, 5, and 6) could be attributed to overlapping specificity and affinity to the agonist and to physical interaction between the receptors, the impact observed on the responsiveness to cytosolic TLRs (TLR-3, 7, and 9) is intriguing. Interestingly, Mukhopadhyay *et al*. utilized deficient mice to demonstrate that macrophage SR-AI/II and MARCO recognize and mediate rapid internalization of agonists to endosomal TLR-3 and cytosolic NOD2 (nucleotide-binding oligomerization domain) and NALP3 (NACHT domain-, leucine-rich repeat-, and pyrin domain-containing protein 3) to elicit robust macrophage responses. Conversely, SR-AI/II and MARCO also internalize TLR-4 ligands, thus attenuating TLR4-mediated responses [14]. Our observations could also be due to an effect of MARCO on mechanisms inherent to cytosolic TLR function. In fact, TLR-3, 7 and 9 must traffic from the endoplasmic reticulum (ER) to endolysosomes before responding to ligands. This trafficking is facilitated by UNC93B1, a multi-pass transmembrane protein localized to the ER [34], [35]. UNC93B1 is not required for responses by surface localized TLRs such as TLR2 and TLR4 [36]. Although our work does not address these two mechanisms in DC, it remains a plausible explanation for the observed MARCO’s wide effects that span the entire TLR family.

Although this work highlights the role of MARCO in DC activation induced by single microbial compounds, we recognize that pathogens express several TLR agonists that may concomitantly engage more than one TLR. For example, selected combinations of TLR agonists have been shown to polarize T cells towards a Th1 phenotype [37]. In such a scenario, one could anticipate that MARCO’s role would be even more significant as it interacts and interferes with signals elicited by various TLRs.

Collectively, our gene expression profiling of MARCO sufficient and deficient mature DC and RT-PCR validation efforts identify a prominent involvement of MARCO in TLR-induced inflammatory responses. MARCO's role seems to span all TLRs, suggesting its implication in the upstream arm of TLR signaling cascade. These new findings add to our understanding of the nuances of DC function in the context of immune regulation by TLR and other pattern recognition receptors. The wide range of genes, pathways and functions that are affected by MARCO in DC warrants more focused future investigation, and opens the prospect of therapeutically targeting MARCO receptor in the hope of ameliorating autoimmune disease, infections and cancer immunotherapy.
